# Exploring prevalence and key determinants of developmental difficulties among young children: a cross-sectional study in urban Bangladesh

**DOI:** 10.1186/s12887-026-06818-2

**Published:** 2026-05-08

**Authors:** Rebeka Jesmin Sarker, Md Biplob Hossain, Jenifar Sarker, A. B. M. Alauddin Chowdhury, Md Jahirul Islam, Md Imdadul Haque

**Affiliations:** 1 Department of Public Health, Faculty of Health and Life Sciences, Daffodil International University, Daffodil Smart City, Birulia, Savar, Dhaka, 1216 Bangladesh; 2 Department of Public Health, Faculty of Health, Education and Life Sciences, Birmingham City University, Birmingham, UK; 3 Department of Family Medicine and Public Health, Spencer Fox Eccles School of Medicine, University of Utah, Salt Lake City, UT USA

**Keywords:** Bangladesh, Developmental difficulties, Urban areas, Young children

## Abstract

**Background and aims:**

Developmental difficulties (DD) refer to delays, disorders, or disabilities in a child’s development across domains such as cognition, language, motor, and social-emotional functioning. Evidence on DD among very young children in urban Bangladesh remains limited. This study aimed to assess the prevalence of suspected DD and identify associated factors among young children in urban Bangladesh.

**Methods:**

This cross-sectional study, conducted in Bangladesh, recruited 405 children aged 0–3 years and their parents using multistage sampling with random ward selection followed by household recruitment. Participants were enrolled from eight different wards in Dhaka City between July 2023 and October 2024. Data were collected through interviews and observations using a semi-structured questionnaire, including the Trivandrum Developmental Screening Chart (TDSC) for 0–3 years. The chi-square test was used to examine the association between the dependent variables and covariates, while logistic regression assessed the strength of the associations.

**Results:**

Overall, 27.9% screened positive for suspected DD. Higher prevalence was observed among younger children (≤ 12 months, *p* = 0.007), children with low birth weight (LBW) (*p* < 0.001), and those from lower- and middle-income families (*p* = 0.025). In adjusted analysis, age ≤ 12 months (AOR: 2.79, 95% CI: 1.65–4.70), LBW (AOR: 6.20, 95% CI: 2.84–13.52), and poor hygiene practices (AOR: 2.83, 95% CI: 1.39–5.75) were independently associated with suspected DD.

**Conclusion:**

One in four young children aged 0–3 years in urban Bangladesh screens positive for suspected DD. The findings underscore the need for early screening and for strengthening clinical, social, and environmental interventions to mitigate developmental challenges and enhance early childhood development.

## Introduction

The first five years of human life, including the 1,000-day window period, are critical for the development of the children’s body organs, brain, nervous system, motor skills, and psycho-social functions [1–5]. Meanwhile, this period represents the most vulnerable phase for the occurrence of DD [4–7]. Hence, the American Academy of Pediatrics recommends periodic screening of children for DD at 9, 18, and 30 months of age with standardized tools [8].

DDs are the altered features of child development, including cognitive, language, emotional, behavioral, social, fine, and gross motor developmental delay [2, 9]. The DDs in early childhood represent a critical public health challenge, significantly influencing morbidity, disability, and quality of life in later stages of life [2]. Worldwide, nearly 316.8 million children and adolescents experience developmental disabilities ranging from mild to severe, including DD. Of which 58.9 million (7.5%) are under-five children (U5C), and the South-East Asia region bears approximately 15% of the global burden [9, 10]. An additional 250 million U5C have been reported to be at risk of developmental crisis in low- and middle-income countries (LMICs) due to poverty, malnutrition, and insufficient parental support [11].

Evidence from multiple indicator cluster surveys (MICS) 2017–2023 showed that 3.4% of children 2–4 years of age in Bangladesh exhibited disabilities or functional difficulties [12, 13]. On the contrary, according to data from a global study across 63 LMICs, approximately 35.4% of Bangladeshi children aged 2–9 have suspected DD, as measured by the Early Childhood Development Index (ECDI). The study also revealed that nearly 7.3%, 31.3%, 12.0%, and 79.6% of Bangladeshi children had exhibited delays in physical, social-emotional, learning, and literacy-numeracy areas, respectively [14]. Another study revealed a 17.1% of U5C in Bangladesh have DD [15]. A study conducted in rural areas of Bangladesh reported DD in 2.6/1000 children and cerebral palsy in 5.6/1000 children aged 18–36 months [16]. However, none of these estimates represent the true prevalence of DD in Bangladesh. Moreover, DDs remain underrepresented among urban young children, where unplanned urbanization, socio-economic disparities, and maternal caregiving challenges pose unique risks to early childhood development [17–19].

Children younger than five had a ten times higher probability of having a disability than of under-5 mortalities [20]. In low-resource settings, factors such as micronutrient deficiencies, inadequate parenting [21], and low birth weight (LBW), maternal education [22], are strongly associated with DD, yet these issues are often overlooked. Additionally, Bangladesh has faced challenges from a high burden of stunting and undernutrition in its U5C since decades [12, 23, 24]. Previous studies have also identified that stunting often starts in utero and extends up to 2 years after birth, negatively impacting children’s physical growth, neurodevelopment, and cognitive functions, with long-term consequences [25]. Further, a national representative survey in Bangladesh showed that stunted children aged 2–4 years old have a significantly increased risk of functional difficulty [26]. However, screening and detection rates are inadequate, leaving many children without timely interventions [27].

The lack of robust epidemiological studies on DD in urban Bangladesh highlights a critical knowledge gap. Additionally, limited awareness and inadequate access to healthcare services in urban areas, especially for the lowest income quantile [17, 19], contribute to delayed detection and management of developmental challenges [12, 28]. Addressing these issues is essential for minimizing the developmental gap and promoting equitable health outcomes. Yet, existing measurement tools are often expensive, complex, or unsuitable for widespread use [27]. This study aims to address these gaps by exploring the prevalence and key determinants of DD among children aged 0–3 years in urban Bangladesh. Understanding these challenges in this vulnerable population is essential for informing evidence-based policies and interventions.

## Materials and methods

### Study design

A cross-sectional research design [16, 18, 22, 29] was employed to estimate the prevalence of suspected DD and to examine the associated factors. The study included young children aged 0–3 years and their parents living in urban areas of Dhaka, Bangladesh. Data collection took place from July 2023 to October 2024. Both mothers and fathers were eligible if they consented to participate. Exclusion criteria included parents of children who were unavailable during the visit, parents who were severely ill, and those who declined to participate.

### Sampling for the study

The sample size was determined using the formula *n* = (z²pq/d²) × (design effect), based on a 95% confidence level, a 17.1% prevalence of DD in U5C in Bangladesh [15], and a 5% margin of error. To account for potential non-responses and dropouts during data collection, a 1.85 design effect was applied, resulting in a final sample size of 403, rounded up to 405.

**Fig. 1:**
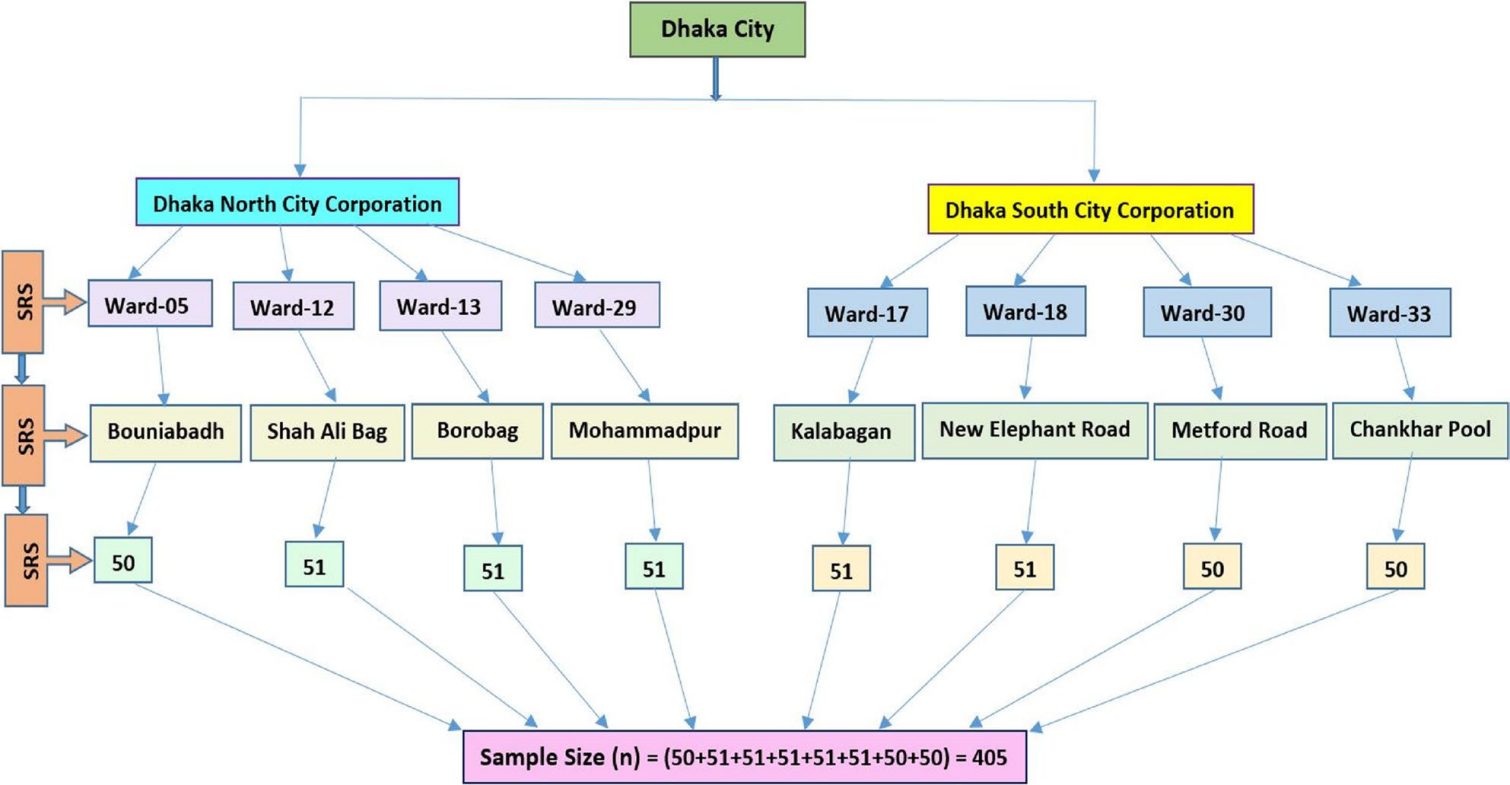
Sample size and sampling strategy of the study

Multistage sampling with random ward selection followed by household recruitment was employed to ensure unbiased participant selection. First, simple random sampling (SRS) was applied to ward lists of Dhaka North City Corporation (DNCC) and Dhaka South City Corporation (DSCC), yielding eight randomly selected wards (four wards from each city corporation) (Fig. 1). Second, one area per ward was randomly selected to represent the diverse urban settings of Dhaka. This two-stage SRS process enhanced the geographical representation of the study population. In the third and final stage, random household visits were conducted within the previously selected areas to recruit eligible children aged 0–3 years and their parents. This practical adaptation ensured the recruitment of 405 participants, maintaining the integrity of randomization. The choice of SRS was justified because it ensured that all eligible participants in the selected urban areas of Dhaka had an equal chance of being selected. This minimized selection bias and facilitated the inclusion of diverse urban contexts, such as slums and residential areas. Given the lack of a pre-established sampling frame and the study’s logistical constraints, an SRS provided a systematic, unbiased approach to participant recruitment.

### Ethics approval

The study was reviewed and approved by the Institutional Review Board and Research Ethics Committee of the Faculty of Health and Life Sciences, Daffodil International University, with ethical clearance number FAHSREC/DIU/2024/SMIG-10. All procedures were conducted in compliance with the ethical standards of the responsible committees on human subjects (institutional and national) and with the Helsinki Declaration of 1964 and later versions. Written informed consent was obtained from all participating parents, with an impartial witness present for illiterate respondents. A Bengali script consent form was used. Before the interview, participants were informed about the study’s background, objectives, and procedures. Anonymity and confidentiality were maintained throughout the process to protect participants’ rights and ensure their safety.

### Data collection and study measures

Before data collection, questionnaires were pre-tested with 10 non-sampled participants to assess their acceptability and relevance. The questionnaire was developed with the guidance and contributions of expert epidemiologists, public health specialists, and statisticians from Bangladesh to serve different study objectives. These experts examined the validity, reliability, and acceptability of the questions for finalization and co-authored this study. Data collectors were extensively trained in the TDSC administration, and inter-observer reliability was ensured through pre-test competency assessments. Data collection was conducted through face-to-face interviews and observation of urban study participants, using a pre-designed semi-structured questionnaire that included the Trivandrum Developmental Screening Chart (TDSC) for 0-3-year-olds. Documentation tools and accessories (a red pen, a bell, a rattle, soft balls, and dolls) were used during data collection. The authors supervised data collection, and parents of children who tested positive for screening (suspected DD) were advised to contact pediatricians at nearby public facilities for further assessment. However, clinical confirmatory follow-up was not conducted due to logistical limitations in this study.

The key determinants and sociodemographic measures observed in this study were family type, monthly family income, socioeconomic class, parents’ educational attainment and occupation, and child’s age and gender. The second critical aspect was pregnancy- and childbirth-related measures, including the child’s birth order and spacing, place of delivery, gestational duration, birth weight of young children, maternal age at childbirth, maternal food satisfaction during pregnancy, and maternal unhappiness during pregnancy. Maternal unhappiness during pregnancy was assessed retrospectively using a self-reported single structured question: Had you gone through unhappiness during your pregnancy period? Responses were recorded as yes or no. This variable reflects perceived maternal affect rather than a clinically diagnosed psychological state. Additionally, other crucial measures related to young children included the adequacy of daily sleep, parental satisfaction with child feeding and hygiene practices, the child’s exposure to environmental risks, and parental relationships. Socioeconomic status (SES) was categorized based on household income quantiles: <12,900 Bangladeshi Taka (BDT) for the lower level, BDT 12,900–21,500 for the middle level, and BDT > 21,500 for the upper level [30]. Further, according to the WHO recommendations, adequate sleep hours for a child were defined as 0–3 months = 14–17 h, 4–11 months = 12–16 h, 1–2 years = 11–14 h, and 3 years = 10–13 hours [31].

The TDSC for 0–3 years was used in this study because it is recognized as simple, convenient, and effective, and the researchers validated it for assessing the DD of young children. The tool is designed for early identification of children at developmental risk, where maximum sensitivity (~ 84%) is critical to avoid missing affected children [29, 32].

TDSC consists of 27 items/questions, applied consecutively as age advances. Each item in the TDSC is age-anchored to normative data, with the cutoff corresponding to the age at which 97% of children in the reference sample successfully achieved the item. Failure to attain any of the item that lies to the left of the child’s chronological age indicates an atypical developmental performance rather than normal variation [22, 29, 32]. For example, the ‘social smile’ function should be exhibited from the first day of birth up to two months of age. Accordingly, the functions such as ‘transfer object hand to hand’ and ‘walk with help’ should be displayed in four months three days to seven months and seven months twenty-four days to 13 months, respectively [22, 29, 32]. Thus, using a one-item delay threshold can effectively identify early delays, which is crucial for children aged 0–3 years [29]. Further, the validation studies also reported a high specificity (> 90%) of the TDSC, indicating a low false-positive rate and confirming the effectiveness of the one-item cutoff for screening in community settings [22, 29, 32].

### Data processing and analysis

The Statistical Package for the Social Sciences (SPSS), Version 22 (IBM Corp., Armonk, NY, USA), was used for data analysis. The final data set was prepared after cleaning, coding, and entering the collected data into the database. Descriptive analysis was performed for all relevant variables to identify frequencies, percentages, and means with standard deviation for normally distributed continuous variables. The chi-square test was used to assess associations between DD and basic categorical descriptors. An independent t-test was used for continuous variables. Only significantly associated (*p* < 0.05) factors in the chi-square test were considered as independent variables in both bivariate and multivariate logistic regression models. The study group was designated as “1” and the reference group as “0” in a binary logistic regression model, which was used to estimate the strength of association through odds ratio computations (crude odds ratio [COR] and AOR), with a 95% confidence interval.

## Results

A total of 405 children aged 0–3 years (mean: 21.19 ± 12.54 months) were included in this study. A significant number of children had illiterate parents (10.6–12.1%), out-of-home-working mothers (42.0%), lower-middle SES (39%), were third-born (15.3%), and were LBW (10.9%). Mothers’ sole responsibility for childcare (87.7%), poor hygiene practices for children (14.8%), parental dissatisfaction with child feeding (15.8%), self-reported environmental risks, such as pollution (51.6%), household oppression (8.6%), and unhealthy parental relationships (9.1%) were also notable (Tables 1 and 2).

**Table 1:** Participants sociodemographic characteristics

Variable	Suspected developmental difficulties*				Sub-total of sample (*n* = 405)		*p*-value for χ^2^**
	Yes (*n* = 113)		No (*n* = 292)				
	Frequency	%	Frequency	%	Frequency	%	
Age of a young child							
Mean ± SD (95% CI)***	21.19 ± 12.54 (19.96–22.42)						0.006
≤ 12 months	48	38.4	77	61.6	125	30.9	0.007
13–24 months	33	22.6	113	77.4	146	36.0	
25–36 months	32	23.9	102	76.1	134	33.1	
Gender of a young child							
Male	70	31.8	150	68.2	220	54.3	0.055
Female	43	23.2	142	76.8	185	45.7	
Family type							
Nuclear	68	27.4	180	72.6	248	61.2	0.786
Joint	45	28.7	112	71.3	157	38.8	
Mother’s education							
Illiterate	18	41.9	25	58.1	43	10.6	0.031
Primary and above	95	26.2	267	73.8	362	89.4	
Father’s education							
Illiterate	17	34.7	32	65.3	49	12.1	0.258
Primary and above	96	27.0	260	73.0	356	87.9	
Mother’s occupation							
Homemaker	75	31.9	160	68.1	235	58.0	0.034
Working	38	22.4	132	77.6	170	42.0	
Monthly family income							
Mean ± SD (95% CI)***	34093.09 ± 24241.37 (31725.09-36461.08)						0.012
Socioeconomic class							
Lower class (< 12900 BDT)	11	33.3	22	66.7	33	8.1	0.025
Middle class (12900–21500 BDT)	45	36.0	80	64.0	125	30.9	
Upper class (> 21500 BDT)	57	23.1	190	76.9	247	61.0	

*SD* Standard deviation, *BDT *Bangladeshi Taka

*Row percentage used

**Level of significance: *p* < 0.05

***Independent t-test

**Table 2:** Factors related to maternal, childbirth, childcare, and household environment

Variable	Suspected developmental difficulties*				Sub-total of sample (*n* = 405)		*p*-value for χ^2^**
	Yes (*n* = 113)		No (*n* = 292)				
	Frequency	%	Frequency	%	Frequency	%	
Birth order							
First	56	26.8	153	73.2	209	51.6	0.824
Second	40	30.1	93	69.9	133	32.8	
Third and above	17	27.4	45	72.6	62	15.3	
Birth Spacing							
Adequate (≥ 2 years)	51	28.3	129	71.7	180	44.4	0.645
Inadequate (< 2 years)	6	37.5	10	62.5	16	4.0	
Not applicable (for first baby)	56	26.8	153	73.2	209	51.6	
Place of delivery							
Institution	96	27.8	250	72.2	346	85.4	0.866
Home	17	28.8	42	71.2	59	14.6	
Gestational duration							
Term (37–42 weeks)	106	27.2	284	72.8	390	96.3	0.099
Preterm (< 37 weeks)	7	46.7	8	53.3	15	3.7	
Birth weight of a young child							
Normal (≥ 2.5 kg)	81	22.4	280	77.6	361	89.1	< 0.001
Low birth weight (< 2.5 kg)	32	72.7	12	27.3	44	10.9	
Maternal age at delivery							
> 19 years	102	27.1	274	72.9	376	92.8	0.211
≤ 19 years	11	37.9	18	62.1	29	7.2	
Mother’s meal intake satisfaction during pregnancy							
Yes	78	23.3	257	76.7	335	82.7	< 0.001
No	35	50.0	35	50.0	70	17.3	
Mother’s unhappiness during pregnancy							
No	43	19.5	178	80.5	221	54.6	< 0.001
Yes	70	38.0	114	62.0	184	45.4	
Parental involvement in child care							
Mother	105	29.6	250	70.4	355	87.7	0.045
Father	8	16.0	42	84.0	50	12.3	
Hygiene practices for young children							
Yes	82	23.8	263	76.2	345	85.2	< 0.001
No	31	51.7	29	48.3	60	14.8	
Parental satisfaction with child feeding							
Yes	86	25.2	255	74.8	341	84.2	0.005
No	27	42.2	37	57.8	64	15.8	
Child sleeps adequately daily.							
Yes	93	25.3	274	74.7	367	90.6	< 0.001
No	20	52.6	18	47.4	38	9.4	
Environmental risk exposure (pollution/oppression)							
No	30	18.3	134	81.7	164	40.5	< 0.001
Yes	83	34.4	158	65.6	241	59.5	
Pattern of environmental risks							
Pollution (air/water/soil/sound)	64	30.6	145	69.4	209	51.6	< 0.001
Oppression (by family)	19	54.3	16	45.7	35	8.6	
Selected risks absent	30	18.6	131	81.4	161	39.8	
Parental Good relationships							
Yes	93	25.3	275	74.7	368	90.9	< 0.001
No	20	54.0	17	46.0	37	9.1	

*SD* Standard deviation

*Row percentage used

**Level of significance: *p* < 0.05

**Fig. 2:**
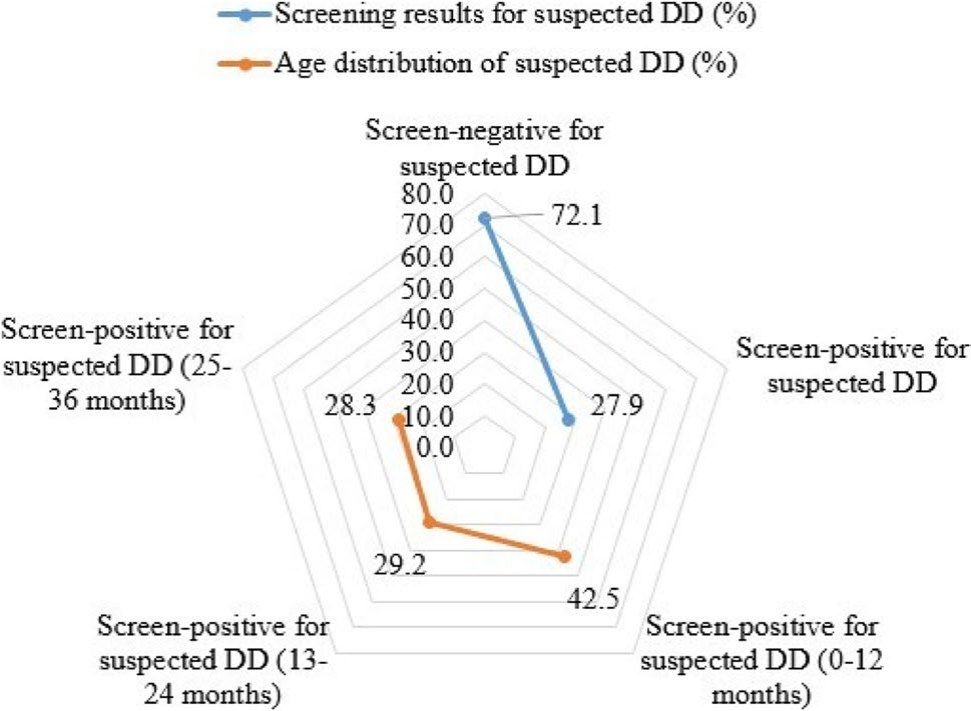
Prevalence of suspected DD among young children aged 0-3 years

Among the children, 113 (27.9%) screened positive for suspected DD. The prevalence was higher among younger children (≤ 12 months, *p* = 0.007) and among children with low birth weight (72.7% vs. 22.4%, *p* < 0.001). Among screen-positive children, 42.5% were aged 0–12 months, 29.2% were 13–24 months, and 28.3% were 25–36 months, indicating a declining trend with advancing age. Higher prevalence of DD was also common among children of mothers with no formal education, homemakers, and those reporting dissatisfaction with meal intake or unhappiness during pregnancy (all *p* ≤ 0.031). Lower- and middle-income SES was also associated with higher prevalence (*p* = 0.025) (Tables 1 and 2; Fig. 2).

Care-related and environmental factors showed a strong association, including poor hygiene practices, children’s inadequate sleep, parental dissatisfaction with child feeding, exposure to environmental risks (pollution or family oppression), and unhealthy parental relationships (all *p* ≤ 0.005). Other variables were not significantly associated with suspected DD. 

Table 3 presents factors that could intensify suspected DD in young children. In adjusted analysis, younger child age (≤ 12 months (AOR: 2.79, 95% C.I.: 1.65–4.70; *p* < 0.001), children with LBW (AOR: 6.20, 95% C.I.: 2.84–13.52; *p* < 0.001), and poor hygiene practices for children (AOR: 2.83, 95% CI: 1.39–5.75, *p* = 0.004) were independently associated with suspected DD. Maternal unhappiness during pregnancy showed a borderline association (AOR: 1.66, 95% C.I.: 0.99–2.78, *p* = 0.054). The model showed good fit (Hosmer-Lemeshow, *p* = 0.387) and moderate explanatory power (Nagelkerke R2 = 0.265).

**Table 3:** Binary logistic regression for addressing the factors contributing to suspected DD among young children

Variable	Crude OR (95% CI)	*p*-value	Adjusted OR (95% CI)	*p*-value*
Age of a young child				
≤ 12 months	2.06 (1.30–3.24)	0.002	2.79 (1.65–4.70)	< 0.001
13–36 months	Ref.		Ref.	
Birth weight				
Low birth weight	9.21 (4.54–18.71)	< 0.001	6.20 (2.84–13.52)	< 0.001
Normal birth weight	Ref.		Ref.	
Socioeconomic status				
Lower and middle classes	1.83 (1.17–2.84)	0.007	0.92 (0.53–1.62)	0.792
Upper class	Ref.		Ref.	
Mother’s education level				
Illiterate	2.02 (1.05–3.87)	0.030	1.20 (0.54–2.68)	0.640
Primary and above	Ref.		Ref.	
Mother’s unhappiness during pregnancy				
Yes	2.54 (1.62–3.97)	< 0.001	1.66 (0.99–2.78)	0.054
No	Ref.		Ref.	
Parental satisfaction with child feeding				
No	2.16 (1.24–3.76)	0.006	1.32 (0.63–2.78)	0.456
Yes	Ref.		Ref.	
Child sleeps adequately daily.				
No	3.27 (1.66–6.45)	0.001	1.66 (0.68–4.04)	0.258
Yes	Ref.		Ref.	
Hygiene practices for young children				
No	3.42 (1.95–6.02)	< 0.001	2.83 (1.39–5.75)	0.004
Yes	Ref.		Ref.	
Parental Good relationship				
No	3.47 (1.74–6.92)	< 0.001	1.26 (0.53–2.96)	0.593
Yes	Ref.		Ref.	
Environmental risk exposure				
Yes	2.34 (1.45–3.78)	< 0.001	1.43 (0.80–2.55)	0.216
No	Ref.		Ref.	
Model summary: Hosmer and Lemenshow goodness of fit test *p* = 0.387, Naegelkerke *R*^2^ = 0.265				

*OR *Odds ratio, *C.I. Confidence interval*,* Ref *Referent

*Level of significance: *p*

## Discussion

This is the first study of its kind in Bangladesh that highlights the prevalence and key determinants of DD among children aged 0–3 years. This study showed a 27.9% prevalence of suspected DD. This finding revealed that more than one in four children younger than three could be suspected of having DD in urban areas of Bangladesh. Age ≤ 12 months, low birth weight, and poor hygiene practices for young children were significant determinants of suspected DD.

### Prevalence comparison

The current study showed a high prevalence of suspected DD among children aged 0–3 years old in an urban setting in Bangladesh, remarkably higher than the findings from earlier studies covering the 2–9 age range in the country [12, 13, 15]. Our estimated prevalence of DD is approximately four times higher than the global estimate of 7.5% [9, 10], surpassing India’s 6.6–16.2% [33, 34] and China’s 4.5–12.5% [35], but significantly lower than that of Pakistan’s 37.9% [36] and Nepal’s 56.4% [37]. In addition, our screening result aligns with the overall suspected prevalence in LMIC [14]. However, our suspected prevalence is also significantly higher than the rates (4.4-11.25%) reported in various community-based studies in India using the TDSC [22, 29, 32, 34]. Conversely, a recent Indian study reported that 42.85% of children aged 1–30 months were TDSC-screen-positive, which is nearly 1.54 times higher than ours [38].

The variation in assessment tools, study areas, and age groups may explain the differing prevalence rates. A significantly higher TDSC-screen-positive rate in our study may be attributed to the focus on younger children, as the risk of DD is greater in this age group [4–7, 39]. The adjusted analysis indicated a significant association between age and DD, with 72.7% of DD cases occurring within the first 24 months, consistent with other research findings [5, 37, 40, 41]. Given these findings, regular screening with standardized tools at key developmental milestones (6, 12, 18, 24, 30, and 60 months) is recommended to enable early identification and timely intervention [8, 42].

### Birth weight

This study found that nearly 10.9% of the children had LBW, and most of them screened positive for DD, with birth weight identified as a significant determinant. This finding aligns with previous studies linking LBW to an increased risk of DD [22, 34, 40]. LBW is associated with higher susceptibility to infectious diseases such as acute respiratory infections (ARIs) and diarrhea [43, 44] as well as undernutrition, including stunting, wasting, and underweight [44], all of which are known risk factors for impaired neurodevelopment and functional difficulties [6, 13, 26, 37, 41, 45]. Linear growth failure, particularly stunting, has been linked to long-term cognitive and developmental deficits [25, 45]. Although we were unable to assess the co-occurrence of infectious or anthropometric deficits due to data limitations, further longitudinal studies are warranted to clarify these pathways and underlying mechanisms.

### Childcare and psychological factors

Maternal unhappiness during pregnancy showed a borderline association with suspected DD, aligning with earlier research that connects maternal depression and poor psychological well-being to adverse child developmental outcomes [6, 39]. Positive maternal affect during pregnancy, responsive caregiving, and healthy parent-child interactions are known to promote self-regulation and social-emotional development, particularly in the first three years of life [21, 46–49]. Although a significant portion of the screen-positive Children experienced unpleasant parental relationships and environmental risks, such as pollution and household oppression; our adjusted model fails to show a statistical association with suspected DD. Nevertheless, a supportive family environment and cognitively stimulating interactions remain critical for optimal early childhood development (ECD) [21, 48, 49].

Mothers solely took care of the majority of screen-positive children. In addition, a substantial proportion of mothers were illiterate, employed outside the home, and resided in nuclear families with lower to middle SES, suggesting potentially inadequate caregiving support. Urban working mothers from lower-middle SES may face challenges related to childcare, nutrition, and responsive parenting, which could, in turn, increase the risk of DD^6,11,13,18,21,23,28,43^. Although we observed a binary relationship between maternal education, occupation, sole caregiving responsibilities, SES, and DD, our adjusted model did not show any significant associations. However, these findings underscore the significance of addressing maternal psychological and socioeconomic factors in early childhood development programs.

### Strengths and limitations of the study

This study provides a novel contribution to the literature by exploring the suspected prevalence and factors associated with DD among young children in urban Bangladesh. This population has been relatively underrepresented in previous research. One of the key strengths of the study is its focus on this specific demographic, which addresses a gap in understanding of ECD in low-resource urban contexts. The findings underscore the importance of addressing maternal and environmental factors to improve developmental outcomes for young children in similar settings.

The study faced some limitations. TDSC serves as a screening tool rather than a diagnostic measure and is prone to over-identifying potential developmental delays, particularly when using a one-item delay threshold. Parents of screened-positive children were advised to contact pediatricians at nearby public facilities for further assessment. However, confirmatory diagnostic follow-up was not conducted due to logistical limitations. Therefore, prevalence was based solely on screening results, which may have led to misclassification and limited the ability to estimate the true prevalence of clinically confirmed DD. Additionally, maternal unhappiness during pregnancy was assessed retrospectively using a single-question, self-reported measure of perceived unhappiness, which may be affected by recall bias and limit accurate classification of psychological state. The study’s cross-sectional design limits the ability to establish causal relationships between the identified risk factors and suspected DD. Moreover, despite using a multistage random sampling approach, the absence of a comprehensive sampling frame necessitated door-to-door recruitment within areas, which may introduce selection bias. Further, selecting only one area per ward and restricting the study to urban settings in Dhaka City may limit the generalizability of the findings to broader contexts. While this design offers valuable insights into associations, longitudinal studies would be needed to confirm causal links and explore how developmental challenges evolve.

## Conclusion

Healthy children are essential for a healthy society. In LMICs like Bangladesh, limited resources, facilities, and awareness often prevent recognition of ECD issues, leaving children vulnerable. This study, which collected data from both slums and residential households, highlights significant risk factors contributing to the higher prevalence of suspected DD among young children in urban Bangladesh. Our findings highlight the need for urgent clinical, social, and environmental interventions to mitigate developmental challenges and enhance early childhood development.

## Data Availability

The supportive data of this study are available from the corresponding author upon reasonable request.

## Abbreviations

AOR: Adjusted odds ratio
ARI: Acute respiratory infection
BDT: Bangladeshi Taka
CI: Confidence interval
COR: Crude odds ratio
DD: Developmental difficulties
DNCC: Dhaka North City Corporation
DSCC: Dhaka South City Corporation
ECD: Early Childhood Development
ECDI: Early Childhood Development Index
LBW: Low birth weight
LMICs: Low- and middle-income countries
MICS: Multiple Indicator Cluster Surveys
NDDs: Neurodevelopmental disorders
OR: Odds ratio
Ref: Referent
SD: Standard deviation
SES: Socioeconomic status
SPSS: Statistical package for social sciences
SRS: Simple random sampling
TDSC: Trivandrum Developmental Screening Chart
U5C: Under-five children
